# Is the Association between Age and Fertility Problems Modified by Diet Quality? Findings from a National Study of Reproductive Age Women in Australia

**DOI:** 10.3390/nu14204355

**Published:** 2022-10-18

**Authors:** Nahal Habibi, Kelly A. Hall, Lisa J. Moran, Dandara G. Haag, Allison M. Hodge, Jessica A. Grieger

**Affiliations:** 1 Robinson Research Institute, The University of Adelaide, Adelaide, SA 5005, Australia; 2 Adelaide Medical School, The University of Adelaide, Adelaide, SA 5005, Australia; 3 School of Public Health, The University of Adelaide, Adelaide, SA 5005, Australia; 4 Monash Centre for Health Research and Implementation, School of Public Health and Preventative Medicine, Monash University, Clayton, VCT 3168, Australia; 5 Cancer Epidemiology Division, Cancer Council Victoria, Melbourne, VCT 3000, Australia; 6 Centre for Epidemiology and Biostatistics, Melbourne School of Population and Global Health, The University of Melbourne, Parkville, VIC 3010, Australia

**Keywords:** age, diet quality, reproductive age, Australia, survey, infertility, women

## Abstract

Background: Increasing age is a strong risk factor for infertility, and there is accumulating evidence of the importance of a healthier diet for fertility. Whether a healthier diet modifies the association between increasing age and infertility has not been investigated. This study aimed to (i) examine if better diet quality could help reduce age-related infertility; and (ii) assess whether changes in diet quality over time are associated with fertility problems. Methods: Data were from Surveys 3 and 5 of the 1973–1978 birth cohort of the Australian Longitudinal Study on Women’s Health. Cross-sectional analysis with multivariable generalized linear models were used to examine the association between age and fertility status, adjusted for various confounders. Multiplicative and additive effect modification by diet quality was assessed, with additive effect modification evaluated with the relative risk for interaction (RERI). Results: In total, 3387 women were included from Survey 3 (age range 24–31 years) and 5614 women from Survey 5 (age range 30–38 years); 588 (17.4%) and 1321 (23.4%) self-reported to have fertility problems in the respective surveys. In Survey 3, compared to younger women with a good-quality diet, older women with a poor-quality diet had a 43% increased risk for fertility problems, with risk increasing after further adjustment for BMI (RR: 1.59; 95% CI: 1.07, 2.37) and PCOS (RR: 1.74; 95% CI: 1.15, 2.62). In Survey 5 in younger women (<33.9 years), there was no association between diet quality and risk for infertility problems. The RERI (across different adjusted models) was between −0.08 (−0.70, 0.55) to −0.39 (−1.40, 0.62) in survey 3 and 0.07 (−0.17, 0.31) to 0.08 (−0.17, 0.32) in Survey 5. Conclusions: There is little evidence to suggest effect modification on the effect of age and fertility problems with diet quality.

## 1. Introduction

Infertility is characterised by the inability of couples to establish pregnancy after a year of regular and unprotected intercourse [1]. Worldwide, infertility impacts up to 15% of reproductive-aged couples [2,3], with prevalence rates varying across countries [4]. Data from the Australian Longitudinal Study on Women’s Health (ALSWH) indicate that one-in-six women aged 28–33 years in 2006 had experienced infertility [5], with longitudinal data across 15 years (2000–2015) from the same study showing the cumulative incidence of fertility problems in women at 14.4% [6]. Infertility contributes considerable public health burden including emotional and psychological distress, social stigmatization, and economic burden [3].

Increasing maternal age is a recognised and well-established risk factor for infertility [7], with age 35 years considered advanced maternal age [8]. In a review of natural fertility populations comprising 58,051 women in developed countries, age-related loss of fertility increased from 4.5% at 25 years of age to 20% at 38 years of age [9]. Global data demonstrate fertility rates declining over time, with greatest declines in younger women and in regions of higher income and education [4]. In Australia, the percentage of women having their first child over the age of 30 has risen from 23% in 1991 to 48% in 2016 [10]. Current data suggest several reasons for this with many women deciding to postpone childbearing to pursue a career, not having a partner, or being unaware of the impact of age on fertility [9,10,11].

Although increasing maternal age is a well-known risk factor for infertility, the fact that age cannot be modified supports the need to examine increasing age in the context of modifiable factors. Obesity is a modifiable factor, consistently associated with infertility [12]. Increasing age is associated with increasing body mass index (BMI), with the rise in obesity prevalence most prominent in women of reproductive age [13]. Whether weight loss in the preconception period is critical to pregnancy success is a topic of current debate [14]. Dietary intake is also a modifiable factor. Research investigating nutritional intake and fertility has flourished over the last two decades, indicating a healthier diet during the preconception period is associated with overall improved fertility outcomes [15,16,17]. Healthier dietary patterns characterised by high consumption of beans, wholegrains, vegetables and fruits [18], greater adherence to a Mediterranean diet [19,20], higher consumption of fruits and lower intakes of fast foods [21], and lower intake of sugar sweetened beverages [22] were associated with shorter time to pregnancy, higher fecundability, higher rates of clinical pregnancy and live birth, and a reduced risk of ovulatory disorder infertility. Interestingly, the study by Chavarro et al. found that the association between the “fertility diet” score and ovulatory infertility was not modified by age, parity, or BMI, with diet composition having a greater apparent impact on fertility than BMI [18]. The study by Karayiannis et al. found that in non-obese women aged <35 years, a 5-point increase in the MedDietScore was associated with ~2.7 times higher likelihood of achieving clinical pregnancy and live birth, but not among women ≥35 years [19].

There is limited information examining dietary intakes across women of different ages, with inconsistent evidence for differences in diet quality between younger and older women [23,24]. Furthermore, whether diet quality modifies the association between age and infertility has not been assessed. Therefore, this study aimed to (i) examine if better diet quality could help reduce age-related infertility; and (ii) assess whether changes in diet quality over time are associated with fertility problems. Our hypothesis is that the effects of age on the risk for fertility problems will be less among women with better diet quality.

## 2. Materials and Methods

### 2.1. Study Design

Our primary aim utilises a cross-sectional analysis at Survey 3 and at Survey 5 to determine whether diet modifies the association between age and fertility problems at each survey, with consideration of a range of confounding factors including BMI. Our secondary aim utilises a longitudinal design to explore whether a change in diet quality from Survey 3 to Survey 5 is associated with fertility problems at Survey 5.

### 2.2. Study Population

This study is based on data from the Australian Longitudinal Study on Women’s Health (ALSWH), a prospective cohort study of women’s physical and mental health, psychosocial aspects of health (such as sociodemographic and lifestyle factors), and use of health services [25]. In 1996, three cohorts of young (born 1973–1978, aged 18–23 years; *n* = 14,247), middle-aged (born 1946–1951, aged 45–50 years; *n* = 13,715), and older (born 1921–1926, aged 70–75 years; *n* = 12,432) women were recruited. Women were randomly selected from the national health insurance scheme (Medicare) database which includes women who are permanent residents of Australia, with national recruitment and intentional over-sampling from rural and remote areas [26]. The women completed a mailed survey every three years, with additional details reported elsewhere [25,26]. All subjects gave their informed consent for inclusion before they participated in the study. The study was conducted in accordance with the Declaration of Helsinki, and the protocol was approved by The Human Research Ethics Committees of the University of Newcastle and the University of Queensland.

This study uses data from the cohort of young women who completed survey 3 in 2003 (25–30 years) and Survey 5 in 2009 (31–36 years). Inclusion criteria were women who completed the FFQ and had known fertility status at either survey (*n* = 2169). The fertility status of 5523 women in Survey 3 (61.3%) and 2464 women in Survey 5 (30.2%) was unknown so they were excluded. We then excluded those with an incomplete FFQ (>10% of items missing responses; *n* = 5) or a reported daily energy intake >14,700 kJ/day (3513.4 Kcal/day) or <2100 kJ/day (501.9 Kcal/day) [27] (*n* = 95 in Survey 3 and *n* = 77 in Survey 5).

### 2.3. Exposure Variables

The exposure variable is age in years, split at the median value, and categorised as younger vs. older women. That is, for Survey 3, the median age was 28.2 years (younger age <28.2 years, older age ≥28.2 years); and for Survey 5, the median age was 33.9 years (younger age <33.9 years, older age ≥33.9 years). The potential effect modifier is diet quality, estimated by the Australian Recommended Food Score (ARFS) [28], obtained from version 2 of the Dietary Questionnaire for Epidemiological Studies (DQES), a validated, semi-quantitative food frequency questionnaire [29]. The DQES uses a 10-point frequency option for participants to report their usual frequency of consumption of 74 foods and beverages over the past 12 months. Serving sizes are adjusted using portion size photographs. Respondents are asked further questions about the total number of daily serves of fruit, vegetables, bread, dairy products, eggs, fat spreads and sugars. The ARFS was derived as previously described [28]. For the alcohol variable, however, we modified the score for pregnancy based on Australian alcohol guidelines “To prevent harm from alcohol to their unborn child, women who are pregnant or planning a pregnancy should not drink alcohol” [30]. Therefore, any alcohol consumption scored 0 points and no alcohol scored 1 point. The highest ARFS is a score of 73, with higher scores indicating better diet quality.

### 2.4. Outcome Variable

Women were asked to report their fertility status in each survey using the following question: “Have you and your partner (current or previous) ever had problems with infertility (that is, tried unsuccessfully to get pregnant for 12 months or more)?” Responses included “no, never tried to get pregnant”, “no, had no problems with fertility”, “yes, but have not sought help/treatment” or “yes, and have sought help/treatment”. Women who responded to “no, never tried to get pregnant” were excluded and women who had problems with fertility, with or without seeking treatment were combined as “yes had fertility problems”. Data were binary coded as “fertility problems’ (coded 1) and “no fertility problems” (coded 0). The resulting sample size in Survey 3 was *n* = 3387, Survey 5 *n* = 5614; and 2169 women were included across both surveys.

### 2.5. Confounding Variables

A range of sociodemographic and health variables were collected at each of the surveys and were identified and included, a priori, as confounders, based on directed acyclic graphs. Surveys 3 and 5 collected information on BMI, country of birth (collected from survey 1), education (highest qualification), SEIFA (socio-economic advantage/disadvantage), physical activity (MET/mins/week), sedentary activity (minutes sitting/week), cigarette smoking, household income, irregular monthly periods, total energy intake (kJ/day), alcohol intake (g/day). Survey 5 additionally collected information on vitamin and mineral supplement usage, and Polycystic Ovary Syndrome (PCOS) status. PCOS is an enduring diagnosis, so we extrapolated the diagnosis from Survey 5 to Survey 3 [31].

### 2.6. Statistical Analysis

Descriptive statistics for Survey 3 and 5 are presented as means and standard deviations for normally distributed continuous variables, medians and interquartile ranges for non-normal continuous variables, and frequencies and percentages for categorical variables. Multivariable generalized linear models (GLM) of Poisson regression with robust variance were fitted to examine the association between age and fertility status for Survey 3 and 5, with adjustment for diet quality, as measured by ARFS diet score (dichotomized at ARFS = 39). Unadjusted models were first assessed, and then model 1 adjusted for ARFS diet score and age, then Model 2 was: Model 1 plus country of birth, education level, SEIFA, physical activity, sedentary activity, cigarette smoking, household income, irregular monthly periods, total energy intake, alcohol intake, vitamin/mineral supplements (Survey 5 only), and the interaction between age and diet score; Model 3 was Model 2 plus BMI (kg/m^2^); and Model 4 was Model 3 plus PCOS status. The estimated effects were quantified as risk ratios (RR) and 95% confidence intervals (CI).

Effect modification by diet quality was evaluated on both the multiplicative and additive scales. Effect measure modification evaluates whether the relationship between one exposure varies within strata of another exposure of interest, whereas the joint exposure effect evaluates the combined effect of two exposures of interest [32]. This is distinct to analyses for interaction, where there are two hypothetical interventions under consideration whereby for effect modification there is only one [33]. We present an effect measure modification analysis because our main interest is to examine the protective role of diet quality on the effect of age on fertility problems, and not the combined effect of diet and age. Tests for multiplicative effect modification were assessed by including the cross product of diet quality and age in the models. Additive effect modification was evaluated with the relative risk for interaction (RERI) using the methods described by Knol et al. [34]. A RERI higher than 0 indicates effect modification greater than the expected additive effect; a RERI of 0 suggests no effect modification; and a negative value indicates effect modification in a negative direction. The RERI is interpreted according to the direction of effect measure modification, as opposed to its size, also recommended by Knol and VanderWeele [32]. The 95% CI of RERI was estimated using the bootstrap method [34].

The secondary aim examined the effect of change in diet between Survey 3 and 5 and fertility status in Survey 5, with the ARFS categorised as ‘good-quality diet (≥39)’ or ‘poor-quality diet (<39)’ [35]. Change in diet was categorized as ‘no change—healthy’ (good-quality diet in both Surveys), ‘no change—unhealthy (poor-quality diet in both surveys), ‘diet improved’ (poor-quality diet in Survey 3, good-quality diet in Survey 5) and ‘worse diet’ (good-quality diet in Survey 3, poor-quality diet in Survey 5)’. Models were fitted with the same adjustment covariates as those in the primary analysis. All statistical analyses performed using Stata version 17 (StataCorp, College Station, TX, USA).

## 3. Results

### 3.1. Participants

A total of 3387 women were included in the analysis at Survey 3 (age range 24–31 years) and 5614 women at Survey 5 (age range 30–38 years), of whom 588 (17.4%) and 1321 (23.4%) self-reported to have fertility problems at the respective survey (Table 1). Across both surveys, the mean Australian Recommended Food Score did not substantially change from Survey 3 to Survey 5, increasing by around 3 units. At both surveys, women with fertility problems had a 1–2 unit higher BMI, and there was a higher percentage of women with irregular periods and with PCOS. At Survey 5, energy intake and metabolic minutes of exercise were slightly lower than at Survey 3, but alcohol intake was higher by around 1 g/day. Between 23–29% of women ever smoked in Survey 3, which fell to 12–13% at Survey 5.

### 3.2. Effect Modification of Diet on Age and Fertility Problems

Table 2 reports the unadjusted and adjusted relative risks for diet and fertility problems in younger and older women at Survey 3. The reference group was young women (<28.2 years) with a good-quality diet (≥39 on the ARFS). Compared to the reference group, younger women with a poor-quality diet (<39) had an increased risk for fertility problems from 20–40%; however, the confidence intervals were wide. Older women with a good-quality diet had an adjusted risk of fertility problems of 1.09 to 1.34 across models 1 to 3, but again, the confidence intervals were wide and did not reach statistical significance. Model 4 that additionally included PCOS demonstrated a 1.69 (0.98, 2.91) increased risk for fertility problems. Compared to the reference group, older women with a poor-quality diet had a 43% increased risk for fertility problems, with increasing risk after further adjustment for BMI (RR: 1.59; 95% CI: 1.07, 2.37) and PCOS (RR: 1.74; 95% CI: 1.15, 2.62).

Table 2 also includes the effect measure modification of diet on the effect of age on fertility problems. A RERI of −0.08 to −0.39 suggests a negative effect measure modification on the additive scale; thus, the effect of age and a good-quality diet was lower than the expected sum of the individual effects of age and diet. However, the 95% CIs overlap 0; thus, additive interaction is absent and little evidence of effect modification of diet in women aged 24–31 years.

At Survey 5, compared to the reference group of younger women [<33.9 years] with a good-quality diet, younger women with a poor-quality diet had an unadjusted increased risk for fertility problems by 18% (95% CI: 1.05, 1.32). However, after adjusting for a series of confounders, the effect estimates were small and not significant (Table 3). Compared to the reference group of younger women [<33.9 years] with a good-quality diet, there was no increased risk for fertility problems in the older women, regardless of diet quality.

Table 3 also shows the effect measure modification of diet on the effect of age on fertility problems. A RERI higher than 0 suggests that the effects of the two exposures operating together is higher than that of each added together (the effect measure modification is positive). However, the 95% CIs overlap 0; thus, additive interaction is not supported in women aged 30–38 years.

### 3.3. Dietary Change and Fertility Status

Table 4 shows the frequency and percentage of women in the cohort by change in diet quality and whether they had fertility problems in Survey 5. There were no important differences in the proportion of women who improved or had a decrease in diet quality, or had no change in diet quality, across fertility status groups. There was no evidence that fertility was associated with a change in diet from Survey 3 to Survey 5 (Table 5).

## 4. Discussion

Among a large cohort of Australian women, we examined whether diet modified the relationship between age and infertility, and whether a change in diet impacts fertility. Our results do not support our hypothesis; that is, in both Survey 3 and in Survey 5, there was little evidence that diet quality modified the association between age and fertility problems. Although increasing age (from Survey 3 to Survey 5) was associated with an approximate 5% higher rate of fertility problems, a change in diet quality over this time was not associated with fertility problems at Survey 5.

Among Survey 3 participants who were aged between 24–31 years, independent associations were found, such that older woman (28.2 to 30.8 years) who had a poor-quality diet had higher risk for infertility problems compared to the younger women (24.4 to <28.2 years) with a good-quality diet. At Survey 5 when women were aged 30–38 years, there was no difference in risk for fertility problems by diet quality or age. This was an unexpected finding. At Survey 5, there was an approximate 10% higher percentage of women with a good-quality diet; thus, with increasing age, the importance of a healthier diet may be realised, irrespective of fertility status. Yet, the older women at Survey 5 did appear to drink slightly more alcohol and perform less metabolic minutes of exercise, which are both associated with higher likelihood for infertility [6,36]. Interestingly, the mean BMI among women with and without fertility problems was consistent over the 5-year period from Survey 3 to Survey 5, with the women with infertility problems maintaining the approximate two-unit higher BMI than the women without infertility problems. The combination of some infertility risk factors increasing over time, but others remaining consistent, may partly explain the lack of association in this slightly older age group of women. Nevertheless, our results might suggest a small window of opportunity in women aged 28–31 years to optimise their diet to support fertility, whereas after this age, the current data do not support this. Although many studies have reported on the independent effects of increasing age with infertility, and increasing evidence shows the importance of a healthy diet for reproductive health, qualitative data reveals that women do not have a clear understanding of the age at which fertility begins to decline [37]. There are also no specific recommendations for an appropriate diet to optimise fertility. The knowledge gaps and misconceptions surrounding reproductive health and nutrition, highlights a critical opportunity that is needed to support patient awareness, counselling to support dietary behaviours, and that involves pregnancy planning.

Our findings provide little evidence of effect modification by diet at either survey. Whilst at Survey 3 there was a suggestion of a negative effect measure modification, such that the effect of age and a good-quality diet was lower than the expected sum of the individual effects of age and diet, the wide 95% CIs are consistent with positive effect modification by diet. At Survey 5, the RERI was positive, suggesting that the effect of age interacting with diet quality was higher than the sum of the independent effects of age and diet, but the wide confidence intervals are also consistent with positive effect modification. Thus, we cannot conclude that better quality diet could attenuate the decline in fertility with age over the observed periods. These findings suggest that the effect of a poor-quality diet and older age on fertility may not be as pronounced as expected, but also that a poor diet quality is not appreciably leading to reduced fertility in older women.

Whilst our results are unexpected, they build the foundation for further studies to assess the interactions between age and diet quality. Although the additive effect modification RERI is the more relevant public health measure, and we would theorise that the targeted group for intervention would be older women with a poor-quality diet, in this instance, we cannot confidently draw this conclusion. Importantly, our results do not mean that a good-quality diet is adverse in any way but implies that a good-quality diet does not attenuate the relationship between age and infertility, and that the joint effect of the two exposures is less than their main effects combined. The indication of a positive RERI at Survey 5 supports the promotion of improved diet quality in older women.

Overall diet quality of women was low in both surveys, which is consistent with previous studies reporting that a poor-quality diet is common amongst women of reproductive age [23,38,39,40,41]. Despite the slight increase in proportion of women having a good-quality diet from Survey 3 to Survey 5, there was no evidence that a change in diet, whether that was an improvement or worsening of diet quality over time, was associated with fertility problems. However, we acknowledge that this change in diet quality is very small and may be equivalent to only replacing white bread with brown (whole meal or multigrain) as the usual bread choice. In line with our findings, a review of studies assessing pregnancy intentions and diet or physical activity behaviours in the preconception and antenatal periods found that women intending to become pregnant do not report different preconception dietary or physical activity behaviours compared with women without pregnancy intentions [42]. This reflects the common barriers observed in women of reproductive age to achieve successful health behaviour change [43]. In our study, the lack of association between a change in diet and fertility may also relate to reverse causation. That is, following a diagnosis of fertility problems, women may alter their behaviours towards healthier choices, or their improvement in dietary intake may not be sufficient to alter the effect on fertility problems.

To the best of our knowledge, this is the first study to assess whether better diet quality can reduce the age-related decline in fertility. A strength of this study is the use of data from a nationally representative cohort of Australian women at two different time points, increasing the generalisability of our findings to all Australian women. We used robust statistical measures, including calculation of the RERI, whereby effect measure modification of diet on the additive scale provides a clear comparison of the effect of diet on the association between one of the major risk factors of fertility (i.e., age) and fertility problems among women. Some limitations are also worth mentioning. A validated, semi-quantitative FFQ was used to capture women’s dietary consumption, which was designed for use in the Australian community. Although the ARFS is an acceptable tool for assessing diet quality, for most foods the scoring was based on ‘at least once per week’. Additional response options might be helpful to quantify diet quality more accurately. While the analysis included a large cohort of women, it may not be sufficiently powered to identify interactions; thus, further work in this area, with larger populations and covering wider age ranges, is warranted. Lastly, the current study included self-reported fertility problems and dietary intakes that could be subject to recall bias.

## 5. Conclusions

In conclusion, our findings indicate that diet quality and older age are independently associated with infertility problems at the younger age range of 24–31 years but not at the older age range of 30–38 years. The present observations provide little evidence that better diet quality could attenuate the decline in fertility associated with age. Investigation of other cohorts with longer follow-up times will be helpful to confirm or refute our findings. Intervention studies to assess the effect of improved diet quality in older and younger women and their effect on fertility may also be helpful to better understand the role that diet has in the context of minimising infertility with increasing age.

## Figures and Tables

**Table 1:** Descriptive characteristics of the study population by infertility status in Surveys 3 and 5.

	Survey 3		Survey 5	
	No fertility Problems (*n* = 2799)	Fertility Problems (*n* = 588)	No Fertility Problems (*n* = 4293)	Fertility Problems (*n* = 1321)
Age (years), Median (IQR)	28.1 (26.9–29.2)	28.3 (26.8–29.2)	33.9 (32.6–35.1)	33.8 (32.6–35.0)
Range	24.6–30.8	24.9–30.8	30.4–37.1	31.0–37.3
Age (categorical), *n* (%)				
Younger	1404 (50.2)	280 (47.6)	2111 (49.2)	666 (50.4)
Older	1395 (49.8)	308 (52.4)	2182 (50.8)	655 (49.6)
ARFS, Mean (SD)	28.9 (9.3)	27.8 (9.1)	32.5 (8.8)	31.5 (8.7)
Median (IQR)	29 (22–35)	28 (22–34)	33 (27–39)	32 (25–38)
Range	4–60	4–51	3–63	2–55
ARFS (categorical), *n* (%)				
Good-quality diet (≥39)	440 (15.7)	76 (12.9)	1097 (25.6)	288 (21.8)
Poor-quality diet (<39)	2359 (84.3)	512 (87.1)	3196 (74.4)	1033 (78.2)
BMI kg/m^2^, Mean (SD)	25.5 (5.4)	27.0 (6.8)	25.6 (5.5)	26.6 (6.4)
Missing	646 (23.1)	120 (20.4)	81 (1.9)	24 (1.8)
Energy (kJ/day), Mean (SD)	7397.0 (2413.0)	7269.6 (2510.1)	6985.0 (2272.7)	6918.7 (2270.9)
Alcohol grams/day, Mean (SD)	6.0 (10.0)	6.1 (10.4)	7.6 (11.8)	7.5 (11.8)
Metabolic minutes of exercise, Median (IQR)	550 (200–999)	533.8 (200–1099)	450 (150–999)	400 (117–999)
Time spent sitting (mins/week), Median (IQR)	2040 (1380–2880)	2340 (1680–3360)	1950 (1260–2760)	2220 (1500–3120)
SEIFA, Mean (SD)	977.4 (84.6)	970.2 (81.8)	1006.1 (89.8)	1005.6 (91.8)
Irregular periods last 12 months, *n* (%)				
No	2078 (74.2)	290 (49.3)	3068 (71.5)	730 (55.3)
Rarely	222 (7.9)	49 (8.3)	494 (11.5)	168 (12.7)
Sometimes	271 (9.7)	76 (12.9)	436 (10.2)	166 (12.6)
Often	218 (7.8)	171 (29.1)	218 (5.1)	234 (17.7)
Missing	10 (0.4)	2 (0.3)	77 (1.8)	23 (1.7)
Polycystic Ovary Syndrome, *n* (%)	66 (2.4)	110 (18.7)	87 (2.0)	225 (17.0)
Missing	604 (21.6)	112 (19.0)	363 (8.5)	96 (7.3)
Use vitamins/minerals, *n* (%)			3621 (84.3)	1164 (88.1)
Missing			3 (0.1)	2 (0.2)
Ever smoked, *n* (%)	660 (23.6)	171 (29.1)	562 (13.1)	161 (12.2)
Missing	4 (0.1)	2 (0.3)	3 (0.1)	1 (0.1)
Country of birth, *n* (%)				
Australian born	2465 (88.1)	521 (88.6)	3751 (87.4)	1151 (87.1)
Other English-speaking background	85 (3.0)	19 (3.2)	150 (3.5)	46 (3.5)
Europe	16 (0.6)	2 (0.3)	30 (0.7)	10 (0.8)
Asia	29 (1.0)	5 (0.9)	54 (1.3)	11 (0.8)
Other	19 (0.7)	2 (0.3)	32 (0.7)	3 (0.2)
Missing	185 (6.6)	39 (6.6)	276 (6.4)	100 (7.6)
Highest education, *n* (%)				
Up to year 12	1199 (42.8)	241 (41.0)	984 (22.9)	313 (23.7)
Trade or certificate/diploma	773 (27.6)	199 (33.8)	1131 (26.3)	383 (29.0)
University or higher university degree	728 (26.0)	128 (21.8)	2070 (48.2)	602 (45.6)
Missing	99 (3.5)	20 (3.4)	108 (2.5)	23 (1.7)
Household income updated, *n* (%)				
No income	13 (0.5)	1 (0.2)	11 (0.3)	6 (0.5)
AUD 1–AUD 36,999 annually	711 (25.4)	146 (24.8)	328 (8.0)	79 (6.2)
AUD 37,000–AUD 51,999 annually	591 (21.1)	103 (17.5)	372 (9.1)	121 (9.5)
AUD 52,000 to AUD 77,999 annually	613 (21.9)	139 (23.6)	775 (19.0)	249 (19.6)
AUD 78,000 or more annually	502 (17.9)	124 (21.1)		
AUD 78,000–AUD 103,000 annually			926 (22.7)	275 (21.7)
AUD 104,000 or more annually			1417 (34.7)	459 (36.2)
Don’t know/don’t want to answer	202 (7.2)	44 (7.5)	256 (6.3)	79 (6.2)
Missing	167 (6.0)	31 (5.3)	562 (13.1)	161 (12.2)

ARFS: Australian Recommended Food Score; SEIFA: Socio-economic index for areas.

**Table 2:** Logistic regression models measuring effect modification of age on fertility by diet quality score [Survey 3].

	Younger Age Group			Older Age Group							
	Good-Quality Diet	Poor-Quality Diet		Good-Quality Diet		Poor-Quality Diet					
Cases/Women	37/252	243/1432		39/264		269/1439					
	RR (95% CI)	RR (95% CI)	*p*-Value	RR (95% CI)	*p*-Value	RR (95% CI)	*p*-Value	RERI (95% CI)	*p*-Value	Int * (95% CI)	*p*-Value
Model 1	Reference	1.21 (0.97, 1.51)	0.090	1.09 (0.94, 1.26)	0.257	1.32 (1.01, 1.26)	0.043				
Model 2	Reference	1.25 (0.89, 1.76)	0.193	1.25 (0.81, 1.95)	0.318	1.43 (1.02, 2.01)	0.039	−0.08 (−0.70, 0.55)	0.811	0.91 (0.57, 1.46)	0.699
Model 3	Reference	1.30 (0.87, 1.94)	0.198	1.34 (0.79, 2.25)	0.275	1.59 (1.07, 2.37)	0.023	−0.04 (−0.82, 0.73)	0.910	0.92 (0.53, 1.59)	0.755
Model 4	Reference	1.44 (0.95, 2.18)	0.089	1.69 (0.98, 2.91)	0.058	1.74 (1.15, 2.62)	0.009	−0.39 (−1.40, 0.62)	0.449	0.72 (0.40, 1.27)	0.254

* Int = Multiplicative Effect Modification; Good-quality diet: ≥39 on the Australian Recommended Food Score; Poor-quality diet: <39 on the Australian Recommended Food Score; Model 1: adjusted for ARFS diet score and the interaction between ARFS diet score and age; Model 2: Model 1 plus country of birth (ethnicity), education (highest qualification), SEIFA (socio-economic advantage/disadvantage) physical activity (MET/mins/week), sedentary activity (minutes sitting/week), cigarette smoking, household income, irregular monthly periods, total energy intake (kJ/day), alcohol intake (g/day), vitamin/mineral supplements (survey 5 only). Model 3: Model 2 plus BMI (kg/m^2^); Model 4: Model 3 plus PCOS status.

**Table 3:** Logistic regression models measuring effect modification of age on fertility by diet quality score [Survey 5].

	Younger Age Group			Older Age Group							
	Good-Quality Diet	Poor-Quality Diet		Good-Quality Diet		Poor-Quality Diet					
Cases/Women	150/706	516/2071		138/679		517/2158					
	RR (95% CI)	RR (95% CI)	*p*-Value	RR (95% CI)	*p*-Value	RR (95% CI)	*p*-Value	RERI (95% CI)	*p*-Value	Int * (95% CI)	*p*-Value
Model 1	Reference	1.18 (1.05, 1.32)	0.006	0.96 (0.87, 1.06)	0.402	1.13 (0.97, 1.31)	0.108				
Model 2	Reference	1.06 (0.89, 1.26)	0.492	0.94 (0.76, 1.18)	0.613	1.09 (0.91, 1.29)	0.355	0.08 (−0.17, 0.32)	0.528	1.08 (0.84, 1.39)	0.536
Model 3	Reference	1.06 (0.89, 1.26)	0.505	0.95 (0.76, 1.18)	0.623	1.08 (0.90, 1.29)	0.406	0.07 (−0.15, 0.30)	0.538	1.07 (0.84, 1.38)	0.577
Model 4	Reference	1.07 (0.90, 1.27)	0.440	0.96 (0.77, 1.20)	0.735	1.10 (0.92, 1.31)	0.290	0.07 (−0.17, 0.31)	0.580	1.07 (0.83, 1.37)	0.608

* Int = Multiplicative Effect Modification; Good-quality diet: ≥39 on the Australian Recommended Food Score; Poor-quality diet: <39 on the Australian Recommended Food Score; Model 1: adjusted for ARFS diet score and the interaction between ARFS diet score and age; Model 2: Model 1 plus country of birth (ethnicity), education (highest qualification), SEIFA (socio-economic advantage/disadvantage) physical activity (MET/mins/week), sedentary activity (minutes sitting/week), cigarette smoking, household income, irregular monthly periods, total energy intake (kJ/day), alcohol intake (g/day), vitamin/mineral supplements (survey 5 only). Model 3: Model 2 plus BMI (kg/m^2^) Model 4: Model 3 plus PCOS status.

**Table 4:** Diet quality change (from Survey 3 to Survey 5) by fertility problem status in Survey 5.

	No Fertility Problems at Survey 5	Fertility Problems at Survey 5	Total
	(*n* = 1671)	(*n* = 498)	(*n* = 2169)
Diet quality change			
No change (healthy)	153 (9.2)	44 (8.8)	197 (9.1)
No change (unhealthy)	1204 (72.1)	359 (72.1)	1563 (72.1)
Diet quality improved	207 (12.4)	67 (13.5)	274 (12.6)
Diet quality worsened	107 (6.4)	28 (5.6)	135 (6.2)

**Table 5:** Results from generalised linear models investigating the association between diet quality change and fertility problems.

	UNADJUSTED		MODEL 1		MODEL 2		MODEL 3	
Diet Quality Change Category	RR (95% CI)	*p*	RR (95% CI)	*p*	RR (95% CI)	*p*	RR (95% CI)	*p*
No change (healthy)	Reference	0.468 *	Reference	0.254 *	Reference	0.262 *	Reference	0.413 *
No change (unhealthy)	0.92 (0.76, 1.13)	0.444	0.98 (0.77, 1.25)	0.884	0.98 (0.77, 1.25)	0.856	1.01 (0.79, 1.29)	0.955
Diet quality improved	0.98 (0.77, 1.24)	0.847	1.16 (0.87, 1.56)	0.313	1.15 (0.85, 1.54)	0.360	1.15 (0.85, 1.55)	0.379
Diet quality worsened	0.81 (0.61, 1.08)	0.153	0.86 (0.60, 1.22)	0.390	0.84 (0.58, 1.21)	0.345	0.86 (0.59, 1.25)	0.426

* Overall *p*-value. Model 1: Adjusted for fertility status in survey 3, country of birth (ethnicity), education (Highest qualification), SEIFA (socio-economic advantage/disadvantage), physical activity (MET/mins/week), sedentary activity (minutes sitting/week), cigarette smoking, household income, irregular monthly periods status, total energy intake (kJ/day), alcohol intake (g/day), vitamin/mineral supplements as measured at Survey 5. Model 2: Model 1 plus BMI (kg/m^2^) Model 3: Model 2 plus PCOS status.

## Data Availability

ALSWH survey data are owned by the Australian Government Department of Health and due to the personal nature of the data collected, release by ALSWH is subject to strict contractual and ethical restrictions. Ethical review of ALSWH is by the Human Research Ethics Committees at The University of Queensland and The University of Newcastle. De-identified data are available to collaborating researchers where a formal request to make use of the material has been approved by the ALSWH Data Access Committee. The committee is receptive of requests for datasets required to replicate results. Information on applying for ALSWH data is available from https://alswh.org.au/for-data-users/applying-for-data/ (accessed on 11 March 2022).
